# Root hairs: an underexplored target for sustainable cereal crop production

**DOI:** 10.1093/jxb/erae275

**Published:** 2024-06-19

**Authors:** Ian Tsang, Jonathan A Atkinson, Stephen Rawsthorne, James Cockram, Fiona Leigh

**Affiliations:** NIAB, 93 Lawrence Weaver Road, Cambridge CB3 0LE, UK; University of Nottingham, Plant Sciences Building, Sutton Bonnington Campus, Nottingham LE12 5RD, UK; University of Nottingham, Plant Sciences Building, Sutton Bonnington Campus, Nottingham LE12 5RD, UK; The Morley Agricultural Foundation, Morley Business Centre, Deopham Road, Morley St Botolph, Wymondham NR18 9DF, UK; NIAB, 93 Lawrence Weaver Road, Cambridge CB3 0LE, UK; NIAB, 93 Lawrence Weaver Road, Cambridge CB3 0LE, UK; University of Antwerp, Belgium

**Keywords:** Arabidopsis, crops, gene function, maize (*Zea mays* L.), rice (*Oryza sativa* L.), sustainable food production, wheat (*Triticum aestivum* L.)

## Abstract

To meet the demands of a rising human population, plant breeders will need to develop improved crop varieties that maximize yield in the face of increasing pressure on crop production. Historically, the optimization of crop root architecture has represented a challenging breeding target due to the inaccessibility of the root systems. Root hairs, single cell projections from the root epidermis, are perhaps the most overlooked component of root architecture traits. Root hairs play a central role in facilitating water, nutrient uptake, and soil cohesion. Current root hair architectures may be suboptimal under future agricultural production regimes, coupled with an increasingly variable climate. Here, we review the genetic control of root hair development in the world’s three most important crops—rice, maize, and wheat—and highlight conservation of gene function between monocots and the model dicot species Arabidopsis. Advances in genomic techniques including gene editing combined with traditional plant breeding methods have the potential to overcome many inherent issues associated with the design of improved root hair architectures. Ultimately, this will enable detailed characterization of the effects of contrasting root hair morphology strategies on crop yield and resilience, and the development of new varieties better adapted to deliver future food security.

## Teaser

We review current knowledge of genes controlling root hair development in rice, maize, and wheat, and highlight conservation of gene function between monocots and the model dicot species Arabidopsis.

## Introduction

Throughout the history of modern plant breeding, much effort has been focused on targeting above-ground traits for gains in yield, nutrient content, and plant health. The challenges of assessing root traits in plant breeding have meant that they currently represent a relatively underexploited target to help increase crop productivity (Table 1). With root hairs being involved in a host of root–soil interactions, here we advocate that exploiting knowledge of their control and function will help deliver the next generation of higher yielding, more nutritious, and increasingly resilient cereal crops to underpin global food production under changing future agricultural environments.

**Table 1.** Highlighting the underexplored nature of roots in crop research and development

Search terms	No. of wheat papers	% of total	No. of rice papers	% of total	No. of maize papers	% of total
[*crop*]	84 331	–	119 395	–	91 726	–
[*crop*]+Yield	12 169	14.4	12 963	10.9	12 727	13.9
[*crop*]+Disease	14 456	17.1	20 743	17.4	10 231	11.2
[*crop*]+Roots	4822	5.7	7138	6.0	6187	6.7
[*crop*]+Roots+Nutrient	601	0.7	839	0.7	722	0.8
[*crop*]+Roots+Lateral	152	0.2	446	0.4	365	0.4
[*crop*]+Roots+Seminal	103	0.1	76	0.1	105	0.1
[*crop*]+Roots+Hair	56	0.1	96	0.1	68	0.1

Literature hits associated with wheat, rice, and maize traits based on queries of the PubMed abstract+title database (queried 10 April 2024). [*crop*] indicates the use of either wheat, rice, or maize as a query term.

**Fig. 1.**
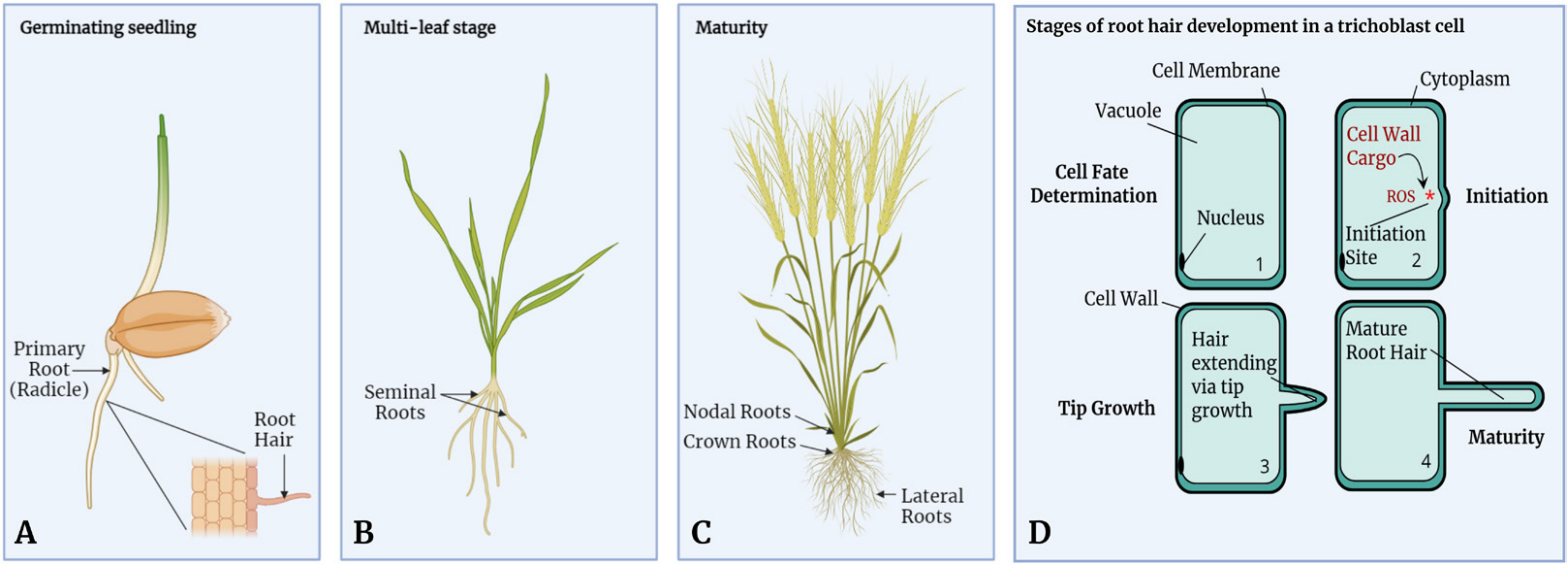
Key features of root architecture in wheat (*Triticum aestivum* L.) at various growth stages. Root hairs are found on all root types. (A) Primary roots and root hairs clearly visible during the seedling stage. (B) Multiple roots form during the multileaf stage. (C) Root system architecture fully established during maturity. (D) The four major stages of root hair development: 1. Cell fate determination: where root epidermal cells have their fate determined to become a hair cell or a non-hair-forming cell. 2. Root hair initiation: an emerging root hair bulge forms as the base of a newly growing root hair. Reactive oxygen species (ROS) production at the bulge aids cell wall loosening; cell wall cargo is transported to the bulge to synthesize the growing root hair. * Illustrates point of initiation. 3. Tip growth: the emerging root hair elongates at the tip. 4. Maturity: the root hair stops growing upon reaching full length.

Globally, food production is dominated by the cereal crops, resulting in an annual harvest of >3 billion tonnes (FAO, 2022). Of these, maize (*Zea mays* L.), rice (*Oryza sativa* L.), and wheat (*Triticum aestivum* L.) represent >90% of the world’s cereal yield (FAO, 2022) and are termed the ‘big three’ global crops. These cereal species all share a series of fundamental root architecture features. First, the primary root (radicle) and seminal roots emerge from the seedling ~1–2 d after germination (illustrated for wheat in Fig. 1) (Ober *et al.*, 2021). The primary root emerges from the basal pole of the embryo (Hochholdinger *et al.*, 2004). In maize, seminal roots (please refer to Table 2 for a glossary of terminology) form from the scutellar node 1 week after germination (Hochholdinger *et al.*, 2004). The coleoptile (shoot emerging from the germinated seed) develops into the primary tiller, with multiple additional tillers developing during the plant’s life. During the adult growth stage, nodal roots develop from all above- and below-ground shoot nodes (Hochholdinger *et al.*, 2004). Crown roots emerge from the below-ground shoot nodes, branching off to form smaller lateral roots. The resulting root architecture enables the plant to capture water and nutrients required for plant growth and development, whilst also providing structural support to help keep the plant upright and avoid lodging.

**Table 2.** A glossary of terms used in this review

Term	Description
Atrichoblast	A root epidermal cell that does not produce root hairs
Cellulose	A large, glucose-based polymer, used to construct plant cell walls
Coleoptile	The developing shoot that emerges from a germinating seed
Crown roots	The roots that emerge from the basal level of the primary tillers in wheat
Cytoskeleton	A complex network of protein filaments that provide structural support and enable intracellular transport
Exocytosis	The process of releasing intracellular cargo through vesicle transport and fusion with the target membrane
Expansins	A group of proteins that regulate plant cell wall modification
Homoeologue	Genes resulting from alloploidy (e.g. in hexaploid bread wheat, three homoeologues are typically present, one on each of the three subgenomes A, B, and D)
Homologue	Genes (and their products) descended from a feature present in a common ancestor, either by speciation or by an intraspecies gene duplication event
Kinase	An enzyme that transfers a phosphate group to a target protein
Nodal roots	The roots that emerge from the first node of the primary tiller in cereal species
Orthologue	As for homologue, but where there is sufficient conservation of surrounding gene order in the chromosomes of the respective species to determine a direct evolutionary descendant from a common ancestor
Peroxidase (PER)	Enzyme that catalyses the breakdown of hydrogen peroxide (H_2_O_2_) to release reactive oxygen radicals (O^−^).
Phosphatase	A protein that removes a phosphate group from a target protein
Reactive oxygen species (ROS)	Intracellular reactive oxygen-containing molecules, such as superoxide (O_2_^−^)
Respiratory burst oxide homologues (RBOHs)	Membrane-bound proteins that catalyse the breakdown of oxygen to form superoxide
Seminal roots	The roots that directly emerge from the germinated seed in wheat
*S*-Palmitoylation	A reversible, post-translational lipid modification step to proteins
Transcription factor	A protein that directly binds and regulates expression of other genes
Trichoblast	A root epidermal cell that produces a root hair
UDP-glucose	A nucleotide sugar
Xyloglucan	A major cell wall hemicellulose

Root hairs develop on the surfaces of all of the different root types (Fig. 1). At the morphological level, plant root hairs are tubular, single-celled outgrowths that emerge from the epidermal cell layer of the root. Root hairs emerge perpendicular to the root surface, and function primarily to anchor the plant root system to a substrate, interact with the rhizosheath, and facilitate nutrient and water uptake (Dolan, 2017; Zhang *et al.*, 2018). In crops, root hairs can enhance stress tolerance and yield stability, and promote shoot phosphate accumulation under drought conditions (Marin *et al.*, 2021). Furthermore, root hair length and densities are increased under low soil phosphate conditions, highlighting the importance of root hairs in crop nutrient uptake (Haling *et al.*, 2013; Nestler and Wissuwa, 2016).

Root hair development can be divided into four broad stages (Fig. 1D). The first stage is cell fate determination, whereby epidermal cells on the emerging radicle (the primary root emerging from the germinated seed) and subsequent roots differentiate into either a hair-forming cell (trichoblast) or a non-hair-forming cell (atrichoblast) (Dolan and Costa, 2001; Grierson *et al*., 2014). Next, ‘bulge initiation’ occurs at a specific site in a trichoblast, where the cell wall forms a bulge as the base of the developing root hair (Grierson *et al*., 2014). The bulge then elongates via a process known as tip growth, until the root hair reaches full length upon maturity (Grierson *et al*., 2014). Notably, the root hairs significantly expand the available surface area for plant roots, occupying up to 50% of the total surface area of a root (Maqbool *et al*., 2022). For example, estimates for the cereal crop species rye (*Secale cereale* L.) indicate that they provide a single plant with an additional 400 m^2^ surface area (Dittmer, 1937). In the cereal crops maize and barley, lack of root hairs results in a clear penalty in field performance (Wen *et al*., 2005; Hochholdinger *et al*., 2008; Zhang *et al*., 2018). Furthermore, longer root hairs are associated with early vigour and rhizosheath size in wheat (Hendriks *et al.*, 2022). Thus, as a critical component of crop root architecture, the modulation of root hair phenotype currently represents an underexploited trait for direct selection in breeding. Despite their importance, much less is known about the genetic regulation of root hair phenotype outside of model plant species such as *Arabidopsis thaliana* L. (subsequently referred to as Arabidopsis). For the first time, this review brings together current knowledge of the genes controlling root hair development in the world’s most important crops: rice, maize, and wheat, and discusses these with reference to homologues in Arabidopsis.

**Fig. 2.**
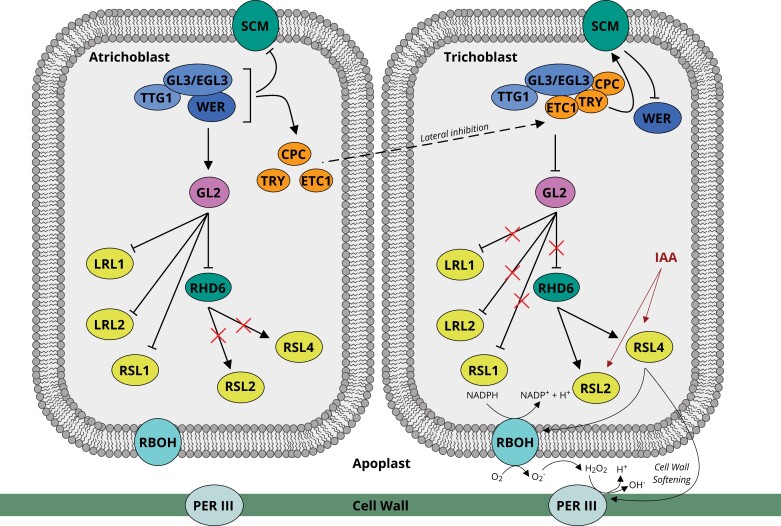
Transcription factors involved in the control of epidermal cell fate determination in Arabidopsis. Solid lines indicate regulation; dashed lines indicate movement. Arrows indicate positive regulation; blunt ends indicate negative regulation. Red crosses indicate disrupted regulation.

Strikingly, while the genes and processes controlling the first two stages of root hair development (epidermal cell fate determination and bulge initiation) are well characterized in the model dicot Arabidopsis, few genes controlling these developmental stages have been identified in rice, maize, or wheat to date. In Arabidopsis, these processes are regulated by a cascade of interacting transcription factors summarized in Fig. 2 (for a recent figurative summary of the genes controlling root hair elongation in Arabidopsis, see Balcerowicz *et al.*, 2015).

Briefly, in non-root hair-forming atrichoblast cells, a trio of transcription factors GLABRA3, ENHANCER OF GLABRA3, and TRANSPARENT TESTA GLABRA1 (GL3, EGL3, and TTG1) form a protein complex known as MBW (Zhang and Hülskamp, 2019), which is activated via binding of the transcription factor WEREWOLF (WER) (Lee and Schiefelbein, 1999). The activated MBW protein complex then mediates downstream signalling cascades within the root epidermal cells that determine epidermal cell fate, achieved via three main pathways (Fig. 2, left). (i) In atrichoblast cells, MBW promotes the expression of *GLABRA2* (*GL2*), which in turn represses expression of the transcription factor gene *ROOT HAIR DEFECTIVE SIX* (*RHD6*), thus modulating the expression of downstream genes that collectively would otherwise lead to differentiation into a trichoblast cell (Menand *et al.*, 2007). (ii) MBW represses expression of the membrane-bound receptor SCRAMBLED (SCM), where SCM would otherwise act to repress *WER* expression (Kwak and Schiefelbein, 2014). (iii) MBW regulates three downstream transcription factor genes *CAPRICE* (*CPC*, Kurata *et al.*, 2005), *TRIPTYCHON* (*TRY*, Schellmann *et al.*, 2002), and *ENHANCER OF TRY AND CPC1* (*ETC1*, Kirik *et al.*, 2004). This trio of transcription factors migrate towards a neighbouring cell to determine trichoblast cell fate (via a lateral inhibition mechanism, Schiefelbein *et al.*, 2014). The arrival of CPC, TRY, and ETC1 at a neighbouring epidermal cell triggers its differentiation into a root hair-forming trichoblast (Fig. 2, right). This is achieved via (i) inhibition of MBW complex activity via competitive inhibition of WER binding (Lee and Schiefelbein, 2002), and (ii) TRY-mediated up-regulation of *SCM*, leading to repression of *WER* transcription (Lee and Schiefelbein, 2002). Collectively, these processes lead to the repression of *GL2*, thus promoting downstream expression of *RHD6, LOTUS JAPONICUS ROOTHAIRLESS1-LIKE 1* (*LRL1*), *LRL2*, *ROOT HAIR DEFECTIVE-SIX LIKE 2* (*RSL2*), and *RSL4* (Menand *et al.*, 2007; Lin *et al.*, 2015), leading to trichoblast bulge initiation.

In the following, we review the genes controlling root hair development in each of the world’s ‘big three’ cereal crops. To aid differentiation of gene nomenclature between the species considered here, we have prefixed gene names with the relevant genus and species names: *Os*, *Zm*, *Ta*, and *At* for rice, maize, wheat, and Arabidopsis, respectively. For the sake of clarity, the genes/gene products discussed in this review have been colour coded in all figures based on biological function. The basic helix–loop–helix (bHLH) transcription factors are coloured pink; reactive oxygen species (ROS) enzymes are coloured red; cell wall modification enzymes are coloured green; vesicle trafficking, cytoskeleton organization, and calcium signalling proteins are coloured blue; and proteins associated with auxin/abscisic acid signalling are coloured orange.

## Rice (*O. sativa*)

**Fig. 3.**
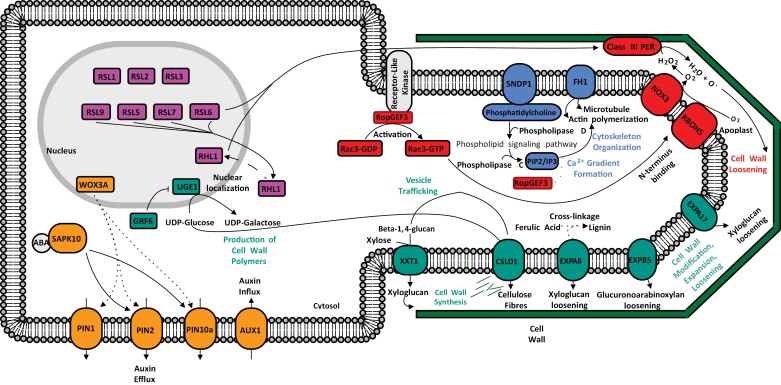
Molecular model for the control of root hair elongation in a rice (*Oryza sativa*) root hair cell (trichoblast). Solid lines indicate regulation, dashed lines indicate movement, dotted lines indicate putative interactions. See main text and Table 3 for details and references.

Of the three cereal crop species focused on in this review, the molecular control of root hair elongation is best understood in rice (Fig. 3).

**Table 3.** Known genes involved in root hair elongation in rice (*Oryza sativa* L.)

Rice gene	Rice gene ID	Functional annotation	Reference
*OsRHL1*	*Os06g0184000*	bHLH transcription factor	Moon *et al.* (2019)
*OsRSL1*	*Os01g0111500*	bHLH transcription factor	Kim *et al.* (2017)
*OsRSL2*	*Os02g0710300*	bHLH transcription factor	Kim *et al.* (2017)
*OsRSL3*	*Os06g0496400*	bHLH transcription factor	Kim *et al.* (2017)
*OsRSL5*	*LOC_Os03g42100*	bHLH transcription factor	Kim *et al.* (2017)
*OsRSL6*	*Os07g0588400*	bHLH transcription factor	Kim *et al.* (2017)
*OsRSL7*	*Os11g0634700*	bHLH transcription factor	Kim *et al.* (2017)
*OsRSL9*	*Os12g0589000*	bHLH transcription factor	Kim *et al.* (2017)
*OsCSLD1*	*Os10g0578200*	Cellulose synthase	Kim *et al.* (2007)
*OsFH1*	*Os01g0897700*	Formin	Huang *et al.* (2013a)
*OsEXPA8*	*Os01g0248900*	Expansin A	Ma *et al.* (2013)
*OsEXPA17*	*Os06g0108600*	Expansin A	ZhiMing *et al.* (2011)
*OsEXPB5*	*Os04g0552200*	Expansin B	Won *et al.* (2010)
*OsWOX3A*	*Os11g0102100, Os12g0101600*	WUSCHEL-related homeobox transcription factor	Yoo *et al.* (2013)
*OsRopGEF3*	*Os02g0272300*	Guanine nucleotide exchange factor	Kim *et al.* (2021)
*OsRac3*	*Os02g0742200*	GTPase	Kim *et al.* (2021)
*OsRBOH5*	*Os05g0528000*	NADPH oxidase	Kim *et al.* (2021)
*OsNOX3*	*Os01g0835500*	NADPH oxidase	Wang *et al.* (2018)
*OsUGE1*	*Os05g0595100*	UDP-galactose/glucose epimerase	Tang *et al.* (2022)
*OsGRF6*	*Os03g0729500*	Growth factor	Yang *et al.* (2023)
*OsXXT1*	*Os03g0300000*	Xyloglucan 6-xylotransferase	Wang *et al.* (2014)
*OsPIN1c*	*Os06g0232300*	Auxin transport	Wang *et al.* (2017)
*OsPIN2*	*Os06g0660200*	Auxin transport	Wang *et al.* (2017)
*OsPIN10a*	*Os01g0643300*	Auxin transport	Wang *et al.* (2017)
*OsSAPK10*	*Os03g0610900*	Stress/ABA-activated protein kinase	Wang *et al.* (2017)
*OsSNDP1*	*Os10g0122600*	Sec14 nodulin protein	Huang *et al.* (2013b)

Rice gene models shown are RAP-DB, apart from MSU gene model *LOC_Os03g42100*.

### RSL Class I transcription factor genes: *RSL1*, *RSL2*, *RSL3*, and *RHL1*

The regulation of epidermal cell fate, hair initiation, and elongation starts in the nucleus. *RSL* (*ROOT HAIR DEFECTIVE SIX-LIKE*) genes encode nuclear-located bHLH transcription factors, which bind and activate expression of root hair-related developmental genes. Expression of *RSL* genes in trichoblasts is critical for wild-type root hair development in Arabidopsis (Balcerowicz *et al.*, 2015). *RSL* genes can be grouped under two categories—Class I and Class II (coloured pink in Fig. 3).

Examples of Class I bHLH genes include *RSL1* (*OsRSL1*), *2* (*OsRSL2*), and *3* (*OsRSL3*) (Kim *et al.*, 2017), and are direct homologues of *RSL* genes identified in Arabidopsis (*AtRSL1* and *AtRHD6*) (Kim *et al.*, 2017). Rice *OsRSL* Class I gene expression is positively correlated with root hair length. In addition, an *osrsl1 osrsl2* double mutant resulted in truncated root hairs, whilst transgenic overexpression of *OsRSL* genes induced root hair formation in atrichoblasts as well as in trichoblasts (Kim *et al.*, 2017). *OsRSL* Class I genes display a degree of functional redundancy, as *osrsl1 osrsl2* double mutants displayed reduced root hair length, while single mutants did not (Kim *et al.*, 2017). Another *RSL* Class I transcription factor gene, *OsRHL1* (*ROOT HAIRLESS 1*), has been found to regulate root hair elongation (Ding *et al.*, 2009, Fig. 3). Similar to *OsRSL* mutants, *osrhl1* mutants also show reduced root hair length. No visual differences in epidermal cell sizes between trichoblasts and atrichoblasts in the *osrhl1* mutant were observed (trichoblasts are normally smaller than atrichoblasts in wild-type plants), suggesting that this gene may play a part in epidermal cell patterning (Ding *et al.*, 2009).

### RSL Class II transcription factor and respiratory burst oxide homologue genes: *OsRSL5*, *OsRSL6*, *OsRSL7*, *OsRSL9*, *OsNOX3, OsRopGEF3*, and *OsRBOH5*

*RSL* Class II genes are also localized to the nucleus and highly conserved across land plants. In total, seven rice *RSL* Class II genes have been identified, of which four show strong expression in root hairs (*OsRSL5*, *6*, *7*, and *9*) (Moon *et al.*, 2019) (Fig. 3, red). These transcription factors bind root hair-specific *cis*-elements (RHEs) within the promoter regions of downstream genes, promoting root hair growth (Moon *et al.*, 2019). Similar to *OsRSL* Class I overexpression, constructs overexpressing *OsRSL5*, *6*, *7*, and *9* individually all had increased root hair length (Moon *et al.*, 2019). Class II *OsRSL* genes were found to guide nuclear localization of the OsRHL1 protein into the nucleus from the cytosol via direct binding with OsRHL1, possibly forming a heterodimer (Moon *et al.*, 2019, illustrated in Fig. 3). Both *OsRHL1* and *OsRSL6* up-regulate Class III peroxidase (PER) genes, which break down hydrogen peroxide (H_2_O_2_) produced from membrane-bound ROS-generating enzymes, enabling cell wall extensibility for subsequent root hair elongation (Fig. 3, red).

ROS-producing enzymes, known commonly as respiratory burst oxide homologues (RBOHs), are widespread throughout plant species. Examples of RBOHs identified in rice include OsNOX3 and OsRBOH5 (Wang *et al.*, 2018; Kim *et al.*, 2021) (Fig. 3, red). RBOHs catalyse the breakdown of O_2_ (oxygen) in the apoplast to O_2_^–^ (superoxide). Superoxide is an unstable intermediate that is rapidly converted to H_2_O_2_ (Nestler *et al.*, 2014). PERs (e.g. Class III PERs) subsequently catalyse the breakdown of H_2_O_2_ to release O^−^ (free oxygen radical), which cleaves the cell wall polymers and enables cell wall extensibility. Cell wall extensibility as a result of RBOH-mediated activity is critical for root hair elongation. Indeed, *osnox3* mutants display reduced root hair length and lower levels of root hair tip intracellular H_2_O_2_ and O_2_^–^ (Wang *et al.*, 2018). In barley (*Hordeum vulgare* L.), a hair-less mutant *rhl1.a* displayed reduced ability to accumulate PER, preventing cell wall loosening and subsequent root hair initiation (Kwasniewski *et al.*, 2013). In legumes, growing root hairs display high levels of ROS in tips, whereby extracellular ATP modulates intracellular ROS production (Cárdenas *et al.*, 2008). Taken together, these results demonstrate the importance of PERs and RBOHs in regulating apoplastic ROS levels and their role in mediating root hair initiation and elongation (see Fig. 3, red).

RBOH activity is regulated through different pathways. In Arabidopsis, auxin-mediated *AtRSL4* expression has been established to regulate ROS generation via interaction with RBOHs and Class III PERs, where RSL4 binds to RHEs of *AtRBOHC* and *AtRBOHJ* (Mangano *et al.*, 2017). In rice, a mechanism regulating RBOH ROS production via guanine nucleotide exchange factors has been identified (Kim *et al.*, 2021). OsRopGEF3 [Rho-of-plant (ROP) protein with Rho guanine nucleotide exchange factor] is a protein localized to the tips of elongating root hairs and facilitates the activation of OsRac3 (ROP Rho-related GTPase). Activation of OsRac3 involves the phosphorylation of OsRac3-GDP to OsRac3-GTP (Kim *et al.*, 2021). The activated OsRac3-GTP interacts with the N-terminus of the membrane-bound OsRBOH5 protein, activating ROS production in the apoplast (Kim *et al.*, 2021) (see Fig. 3, red). The mutant *osropgef3* had reduced root hair length and increased root hair width. The functional interaction between OsRac3 and OsRBOH5 was confirmed by reduced levels of ROS production in *osropgef3* mutant root hairs (Kim *et al.*, 2021). It is speculated that OsRopGEF3 interacts with a plasma membrane protein (possibly a receptor-like kinase) through a PRONE (plant-specific ROP nucleotide exchanger) protein domain on the membrane protein (Kim *et al.*, 2021) (Fig. 3 sections in grey and red). The authors reported that varying auxin levels failed to alter expression of *OsRBOH5*, indicating that it may not be regulated by auxin or *RSL* genes. This indicates regulatory differences of RBOHs between rice and Arabidopsis.

### Cell wall modification genes: *OsCSLD1*, *OsEXPA8*, *OsEXPA17*, *OsEXPB5*, *OsXXT1*, and *OsUGE1*

Cell wall-modifying proteins play a major role in facilitating root hair elongation. As root hairs elongate via extension of the cell wall and membrane, multiple processes, including cell wall softening, expansion, loosening, and synthesis, must be coordinated.

In plants, cell walls can be categorized into two major types—Type I and Type II. Type I cell walls are primarily found in the gymnosperms and dicots, whereas Type II cell walls are common in monocots (Won *et al.*, 2010) such as rice, maize, and wheat. Type I cell walls are primarily formed of a cellulose–xyloglucan (XG) network, rich in pectin and structural proteins (Wang *et al.*, 2014). In contrast, Type II cell walls are rich in cellulose fibres encased in glucuronoarabinoxylan (GAX, a hemicellulose) with comparatively lower levels of pectin, xyloglucan, and structural proteins (Wang *et al.*, 2014). Despite such differences between Type I and II cell walls, many genes regulating cell wall properties in dicots (e.g. Arabidopsis) are highly conserved in monocots, including rice (coloured green in Fig. 3).

Despite the lower XG content (1–5%) in Type II (monocot) cell walls, XG is still a critical component (Wang *et al.*, 2014). The gene *OsXXT1* encodes a root hair-specific, membrane-bound protein [xyloglucan 6-xylotransferase 1 (XXT1)], responsible for catalysing the transfer of xylose to β-1,4-glucan chains (Wang *et al.*, 2014) (Fig. 3, green). A missense point mutation (G 1009A) spanning a highly conserved domain in XXT proteins resulted in shorter root hairs in the *osxxt1/2* mutant, which also displayed slower seedling growth rates (Wang *et al.*, 2014). This illustrates that despite the low XG content in Type II cell walls, XG is still likely to be critical for normal root hair growth in monocot trichoblasts.

Cellulose synthesis is a critical component of cell wall growth, which is a process regulated by cellulose synthases. Cellulose consists of linear β-1,4 glucans that form fibrils. In rice, *OsCSLD1* (cellulose synthase-like D1) encodes a trichoblast-specific cellulose synthase and is a likely functional homologue of Arabidopsis *AtCSLD3* (Kim *et al.*, 2007) (Fig. 3, green). The *oscsld1* mutants have reduced root hair length, and seedlings display truncated seminal root development, indicating that *OsCSLD1* is involved in both root hair and seminal root development (Kim *et al.*, 2007). In addition, *oscsld1* mutants had swellings and bulges at root hair tips, indicating abnormal cell growth (Kim *et al.*, 2007). Seminal roots of *oscsld1* had cells similar in size to the wild type, indicating that the shorter seminal roots are due to lower cell numbers in the *oscsld1* mutant rather than smaller cell sizes. This implies that *OsCSLD1* is a regulator of cell number in rice roots (Kim *et al.*, 2007).

The primary substrate used to synthesize cellulose is UDP-glucose (Tang *et al.*, 2022). The *UGE* (UDP-glucose 4-epimerase 1-like) genes encode enzymes that catalyse the conversion of UDP-galactose to UDP-glucose (or vice versa in a gene/enzyme-dependent manner) (Yang *et al.*, 2023). Four *UGE* genes have been identified in the rice genome (*OsUGE1*, *2*, *3*, and *4*). The isoform of UGE encoded by *OsUGE1* converts UDP-glucose to UDP-galactose, thereby acting as a negative regulator of root hair elongation (Yang *et al.*, 2023) (Fig. 3, green). The mutant *osuge1* lines displayed longer root hairs relative to the wild type, while *OsUGE1* overexpression lines had shorter root hairs (Yang *et al.*, 2023). The authors also identified a role in root hair elongation for OsGRF6 (Growth Factor 6), a *cis*-transcription factor that directly represses *OsUGE1* expression (Fig. 3, green). Through repression of *OsUGE1* activity, more UDP-glucose is present in the cytosol for cellulose synthesis via *CSLD* genes, promoting root hair elongation. In Arabidopsis, *AtUGE4* also acts as a negative regulator of root hair elongation and is a likely functional homologue of *OsUGE1* (Yang *et al.*, 2023).

After cell wall synthesis, expansins (EXPs) are a group of proteins that regulate plant cell wall modification. EXPs are classed into two subfamilies—A (α) and B (β) (Ma *et al.*, 2013). The EXPAs primarily function in the cell walls of dicots, while EXPBs function primarily in cell walls of graminaceous monocots (Ma *et al.*, 2013). As such, EXPAs are more common in dicots, while EXPBs are more common in monocots. EXPs facilitate cell wall extensibility and elongation, possibly via disrupting non-covalent bonds between cellulose fibres and polysaccharides in the cell wall (Won *et al.*, 2010; Ma *et al.*, 2013) (Fig. 3, green). In rice, *OsEXPA8* and *OsEXPA17* are examples of identified α EXP genes. Lines overexpressing *OsEXPA8* had altered cell walls with higher polysaccharide:lignin ratios, indicating reduced cell wall stiffness, which was correlated with increased cell wall extension and cell length in the vascular bundle (Ma *et al.*, 2013). Interestingly, *OsEXPA8* overexpression lines also displayed an increased number of lateral roots and root hairs, indicating that cell growth in both roots and root hairs is regulated by EXPs (Ma *et al.*, 2013). *OsEXPA17* is also a root hair-specific α EXP gene that facilitates cell wall loosening. A point mutation in *OsEXPA17* resulted in shorter root hairs, while overexpression lines displayed longer root hairs (ZhiMing *et al.*, 2011). Two RHEs were also identified in the *OsEXPA17* promoter, driving high expression in the trichoblast (ZhiMing *et al.*, 2011). Rice *OsEXPB5* is a β EXP gene, identified as a homologue of the barley root hair initiation gene *HvEXPB1* (Kwasniewski and Szarejko, 2006). Thus, despite the differences in cell wall composition between Type I and Type II cell walls in dicots versus monocots, it is likely that EXPAs and EXPBs are both necessary for cell wall modification in monocots, as both XG and GAXs are present (Won *et al.*, 2010).

While no studies have currently determined specific differences in the cell wall composition of root hairs between monocots and dicots, we can speculate on some differences via the cellulose synthase (*Csl*) gene family. Csls are a major component of cell walls, responsible for the synthesis of the hemicellulose polysaccharide backbone (Kaur *et al.*, 2017). *CSL* genes span nine subfamilies (Kaur *et al.*, 2017), where monocots lack *CslB* and *CslG* families. *CslF* and *H* families are restricted to grass species, whereas *CslA*, *C*, and *D* are conserved across all land plants. In particular, *CslF* gene products regulate the synthesis of (1,3:1,4)-β-d-glucans (Burton *et al.*, 2011), a characteristic feature of cell walls in cereals, which are less common in dicots (Burton *et al.*, 2011). This highlights the potential differences in cell wall composition in root hairs between monocots and dicots.

### Cytoskeleton organization and calcium gradients: *OsSNDP1* and *OsFH1*

The formation of a functioning cytoskeleton is critical for root hair elongation. The cytoskeleton comprises actin microfilaments, microtubules, and intermediate filaments, which help determine cell polarity by establishing intracellular directional transport (Takatsuka and Ito, 2020). Polarity is further established and maintained via calcium channels regulating cytosolic Ca^2+^ (calcium) (Brost *et al.*, 2019). Furthermore, myosin (a motor protein that converts ATP into mechanical energy) interacts with actin filaments to establish intracellular vesicle trafficking, delivering vesicles containing cell wall polymers to cell wall proteins for cell wall construction (Takatsuka and Ito, 2020). As such, the cytoskeleton, calcium signalling, and vesicle trafficking are critical processes for regulating root hair elongation (genes coloured blue in Fig. 3).

Phospholipid signalling is a process that regulates cytoskeleton organization and vesicle trafficking. Briefly, phosphatidylinositol transfer proteins (PITPs) enable phospholipase D hydrolysis of phosphatidylcholine, releasing phosphatidic acid and phosphatidylinositol 5-kinase, producing phosphatidylinositol 4,5-bisphosphate (PIP2) (Huang *et al.*, 2013b). PIP2 or IP3 (produced via phospholipase C hydrolysis of PIP2) are thought to regulate cytoskeleton organization via actin polymerization, microtubule bundling, and subsequent Ca^2+^ gradient formation (Huang *et al.*, 2013b) (Fig. 3, blue). The *OsSNDP1* gene encodes a rice PITP, with *ossndp1* mutants displaying altered root hair phenotype with branched and domed shapes (Huang *et al.*, 2013b). *OsSNDP1* is a likely homologue of Arabidopsis *AtCOW1*, mutants of which display shorter, branched root hairs (Böhme *et al.*, 2004) as observed in rice. Fusions of green fluorescent protein (GFP) with AtCOW1 revealed that the protein is expressed in root hairs, especially in those undergoing active growth (Böhme *et al.*, 2004).

Cytoskeleton organization is also regulated by formins, a group of proteins responsible for microtubule bundling and actin polymerization, which usually contain a highly conserved formin homology 2 (FH2) protein domain (Huang *et al.*, 2013a) (Fig. 3, blue). A point mutation in the FH2 domain encoded by the gene *OsFH1* resulted in truncated root hairs, indicating that formins are critical for root hair elongation in rice (Huang *et al.*, 2013a). *OsFH1* is expressed across the whole plant from root to shoot, explaining why *osfh1* mutants also displayed reduced shoot length.

### Auxin response: *OsPIN1*, *OsWOX3A*, and *OsAUX1*

Auxins are an extremely well-studied class of plant growth hormone, critical for many biological processes in plants including root hair elongation. In Arabidopsis, root hair elongation in response to auxin has been well characterized. Auxin [in the form of indole-3-acetic acid (IAA)] interacts with auxin response factors (ARFs; transcription factors that regulate downstream auxin-responsive genes) (Vissenberg *et al.*, 2020). For example, auxin is known to regulate ROS production through interaction with bHLH transcription factors, such as AtRSL4 (Mangano *et al.*, 2017).

Auxin transport proteins, including the PIN (PIN-FORMED) and ARF proteins, regulate epidermal cell auxin concentrations (Yoo *et al.*, 2013) (coloured orange in Fig. 3). In rice, expression of these transport proteins is regulated by *OsWOX3A*, a gene encoding the WUSCHEL-related homeobox (WOX) transcription factor (Yoo *et al.*, 2013). The *OsWOX3A* gene has been found to regulate both lateral root development (Cho and Paek, 2016) and root hair elongation (Yoo *et al.*, 2013) (Fig. 3, orange). Rice *oswox3a* mutants had increased root hair length, but grew shorter and fewer lateral roots compared with the wild type (Yoo *et al.*, 2013). Rice *OsPIN1* expression was reduced in the *oswox3a* mutant, indicating that reduced auxin efflux from cells (thereby increasing intracellular auxin concentration) increased root hair growth. This was confirmed through exogenous auxin treatment of the *oswox3a* mutant which increased root hair growth and reduced lateral root count (Yoo *et al.*, 2013). Yoo *et al.* (2013) proposed that reduced auxin transport decreases auxin concentration in the pericycle cells that encircle root vascular tissues, and increases auxin concentration in epidermal cells, thus reducing lateral root development and promoting root hair elongation, respectively. The rice gene *OsAUX1* encodes an auxin influx transporter, which regulates the root angle in rice (Giri *et al.*, 2018) (Fig. 3, orange). The gene *OsAUX1* encodes an auxin transporter, which was found to promote root hair elongation in rice under low soil phosphate conditions (Giri *et al.*, 2018). OsAUX1 transports auxin from the root apex to the root hair elongation zone, promoting root hair elongation in regions of low external phosphate (Giri *et al.*, 2018). This highlights the importance of root hairs in response to phosphate uptake in low phosphate conditions.

### Abscisic acid response: *OsSAPK10*

Abscisic acid (ABA) is another critical plant hormone, primarily controlling abiotic stress responses in plants (Vissenberg *et al.*, 2020). In Arabidopsis, ABA suppresses root hair growth via positively regulating expression of the gene encoding the transcription factor OBF BINDING PROTEIN4 (OBF4), which binds to the *AtRSL2* promoter, repressing expression and inhibiting root hair growth (Rymen *et al.*, 2017). Conversely, ABA has been found to promote root hair elongation in rice through regulating auxin homeostasis (Wang *et al.*, 2017). Rice ABA-insensitive lines displayed shorter root hairs, while ABA-hypersensitive lines (overexpressing *OsSAPK10*, a stress/ABA-activated protein kinase) had increased root hair length (Fig. 3, orange). Exogenous ABA was found to induce ectopic expression of the rice PIN genes *OsPIN2* and *OsPIN10a* in mature root hair zones (Wang *et al.*, 2017) (Fig. 3 section in orange). It is proposed that the ectopic expression of *PIN* genes drives auxin efflux, which then flows towards the regions of elongating root hairs, increasing local auxin concentration and promoting subsequent root hair elongation (Wang *et al.*, 2017).

### Rice summary

Rice root hair development involves the coordination of several major cellular processes. Nuclear transcription factors (e.g. OsRSL1 and OsRHL1) activate root hair-specific genes, triggering cell wall modification (e.g. via EXPs, OsEXPA17; and cellulose synthases, OsCSLD1) and the organization of the cytoskeleton to transport cell wall cargo towards the emerging root hair bulge (e.g. via Rop GTPases, OsRopGEF3; and formins, OsFH1). Additionally, plant hormones such as auxin and ABA regulate root hair growth via interaction with auxin transporters (e.g. OsAUX1 and OsPIN2) and ABA kinases (OsSAPK10). As evident in the next sections, many of the genes involved in these major processes are highly conserved between cereal species and Arabidopsis, allowing homologous genes characterized in rice to be identified in related species such as maize and wheat.

## Maize (*Zea mays*)

In contrast to rice, fewer genes have been identified and characterized to play a role in maize root hair elongation (Table 4). The main family of functionally characterized maize root hair genes are known as the *RTH* (*roothairless*) genes. Here, we discuss their role, as well as that of other known genes in maize root hair elongation.

**Table 4.** A subset of genes involved in root hair elongation in maize (*Zea mays* L.)

Maize gene	Maize gene ID	Functional annotation	References
*ZmRTH1*	*Zm00001eb051220*	Sec3 exocyst	Wen *et al.* (2005)
*ZmRTH3*	*Zm00001eb014120*	COBRA protein	Hochholdinger *et al.* (2008)
*ZmRTH5*	*Zm00001eb148780*	NADPH oxidase	Nestler *et al.* (2014)
*ZmRTH6*	*Zm00001eb025100*	Cellulose synthase	Li *et al.* (2016)
*ZmTIP1*	*Zm00001eb386990*	*S*-Acyltransferase	Zhang *et al.* (2020)
*ZmLRL5*	*Zm00001eb374700*	Nuclear transcription factor	Wang *et al.* (2019)

Maize Gene Model: B73.

### Vesicle transport: *ZmRTH1*

**Fig. 4.**
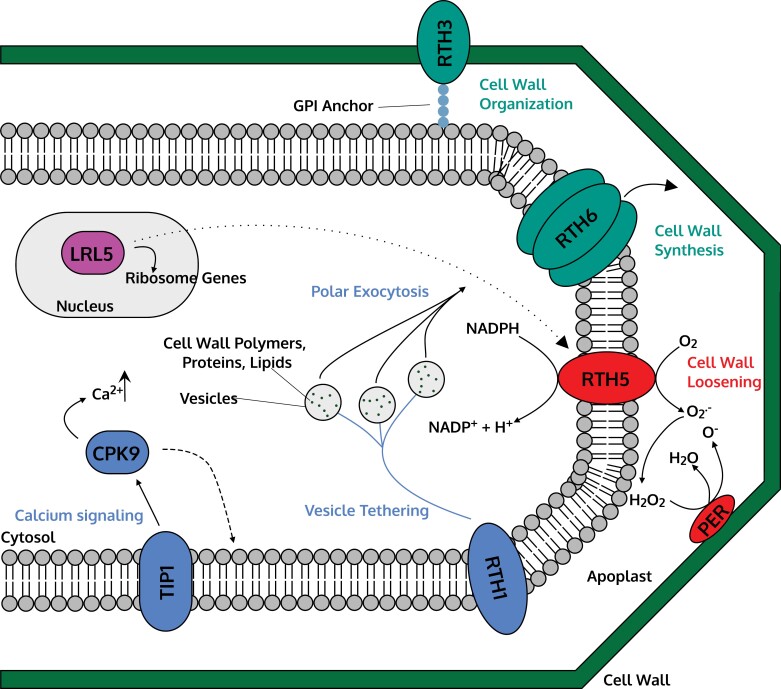
Molecular model for the control of root hair elongation in a maize (*Zea mays*) root hair cell (trichoblast). Solid lines indicate regulation, dashed lines indicate movement, and dotted lines indicate putative interactions. See main text and Table 4 for details and references.

The first maize *RTH* gene was identified by Wen *et al.* (2005) through the *rth1* mutant. While this line displayed bulge initiation, root hair elongation was impaired. The *ZmRTH1* gene linked to the *rth1* mutant phenotype was identified as a close homologue of *SEC3* (*Subunit of the exocyst complex 3*) from yeast (*Saccharomyces cerevisiae*) (Wen *et al.*, 2005). *SEC3* and *RTH1* both encode an exocytotic complex that associates with the plasma membrane and is responsible for regulating polar exocytosis (fusion of vesicles with the plasma membrane). In maize, this process involves the transport of vesicles containing cell wall materials (proteins/polymers) towards the site of root hair elongation (Fig. 4, blue). Interestingly, the maize *rth1* mutant exhibited significant phenotypic defects when grown in the field, including stunted plant height and failure to develop ears (Wen *et al.*, 2005). This indicates that *RTH1* may be involved in multiple developmental roles in maize.

### Cell wall modification: *ZmRTH3* and *ZmRTH6*

Maize *rth3* mutants display a similar phenotype to *zmrth1*, forming bulge initiations on the root surface, but failing to elongate properly into root hairs (Hochholdinger *et al.*, 2008). *RTH3* encodes a COBRA-like protein [which functions as a glycosylphosphatidylinositol (GPI) anchor], highly expressed in both developing trichoblasts and lateral root primordia (Hochholdinger *et al.*, 2008). Within the plant COBRA protein family, the Arabidopsis homologue (AtCOBRA) aids cellulose microfibril deposition during organ expansion (Schindelman *et al.*, 2001). The ZmRTH3 protein probably plays a similar role in root hair elongation in maize, organizing the synthesized cellulose bundles from cellulose synthases (Fig. 4, green). In the field, maize *zmrth3* mutants exhibited a severe grain yield penalty of up to 42.3% compared with wild-type lines (Hochholdinger *et al.*, 2008).

Cellulose synthases construct cellulose strands for the elongating root hair. The product of the maize root hair gene *ZmRTH6* was characterized as a CSLD5 (cellulose synthase-like D5) membrane-bound cellulose synthase (Li *et al.*, 2016). Maize *zmrth6* mutants form bulges but the root hairs fail to extend. The *ZmRTH6* gene is part of a highly conserved clade of D-type cellulose synthase genes, with homologues found in a diverse range of species including the moss *Physcomitrella patens* (Li *et al.*, 2016). This gene is responsible for the synthesis of the α (primary) cell wall layer. In root hairs, the α cell wall layer is a thinner layer deposited at the emerging root hair tip which expands via turgor pressure (Galway, 2006). This layer is crucial for elongation of root hairs. In contrast, the β (secondary) cell layer is a thicker, multicell wall layer, deposited at the base of root hairs for structural support (Galway, 2006; Li *et al.*, 2016). The ZmRTH3 protein probably organizes the synthesized cellulose produced by ZmRTH6, as *ZmRTH6* is significantly down-regulated in both the *zmrth3* and *zmrth5* mutants, indicating clear evidence of interaction between these gene products (Li *et al.*, 2016) (Fig. 4, green).

### ROS production: *ZmRTH5*

As previously described for rice, ROS production is critical for root hair elongation. Nestler *et al.* (2014) identified and characterized the maize gene *RTH5*, encoding a membrane-bound RBOH that produces ROS. The *ZmRTH5* gene is highly expressed in root hairs, and the *zmrth5* mutant has shorter root hairs and reduced root hair density compared with wild-type roots. Maize *ZmRTH5* is a homologue of *OsNOX3* in rice (Wang *et al.*, 2018). *ZmRTH5* is also distantly related to *AtRHD2* in Arabidopsis, which encodes a similar RBOH (RBOHC) (Kuběnová *et al.*, 2022). As described above, RTH5 facilitates cell wall loosening through catalysing the production of superoxide (Fig. 4, red). In maize, ZmRTH5*-*mediated ROS production is also likely to be critical for establishing cell polarity in developing trichoblasts (Mangano *et al.*, 2017).

### Calcium signalling and *S*-palmitoylation: *ZmTIP1*

The root hair elongation gene *ZmTIP1* (*Tip Growth Defective 1*) encodes an *S*-acyltransferase (Zhang *et al.*, 2020). *S*-Acyltransferases regulate the process of *S*-palmitoylation, a reversible post-translational lipid modification step to proteins, where a 16-carbon palmitate (an ester of palmatic acid) is added to a cysteine residue via a thioester bond (Zhang *et al.*, 2020). Maize *ZmTIP1* was identified via a genome-wide association study (GWAS), where it was found to regulate root hair length and drought tolerance in a mapping panel of 166 inbred lines (Zhang *et al.*, 2020). This protein is localized to the plasma membrane and Golgi compartments in trichoblasts, and interacts directly with ZmCPK9 (Calcium-dependent Protein Kinase 9), a calcium protein kinase (Zhang *et al.*, 2020). ZmTIP1 modifies ZmCPK9 via *S*-acyltransferase activity, facilitating CPK9 association with the plasma membrane (Fig. 4, blue). This process is key for maize root hair elongation, probably due to ZmCPK9 maintaining a high local concentration of Ca^2+^ in the root hair apex (Zhang *et al.*, 2020). High Ca^2+^ levels in the expanding trichoblast may also activate RBOHs for ROS production (Nestler *et al.*, 2014).

### Nuclear transcriptional regulation: *ZmLRL5*

Nuclear transcription factors often act as master regulators of gene networks controlling root hair elongation. bHLH transcription factor genes have been well characterized in Arabidopsis (*RSL1*, *RSL2*, *LRL1*, and *LRL2*, Balcerowicz *et al.*, 2015; *RSL4*, Mangano *et al.*, 2017) and rice (*OsRSL1*, *OsRSL2*, and *OsRSL3*, Kim *et al.*, 2017; *OsRHL1*, Ding *et al.*, 2009; *OsRSL5*, *OsRSL6*, *OsRSL7*, and *OsRSL9*, Moon *et al.*, 2019). In maize, a similar bHLH transcription factor gene (*ZmLRL5*) has been identified to play a role in root hair elongation (Fig. 4, pink), with the *zmlrl5* mutant displaying a shorter root hair phenotype compared with the wild type (Wang *et al.*, 2019). *LRL* (*LOTUS JAPONICUS ROOTHAIRLESS1-LIKE*) genes encode bHLH transcription factors and are, along with RSL genes, highly conserved, and responsible for the development of root hairs (e.g. in Arabidopsis) and rhizoids (e.g. in the moss *P. patens*) in land plants (Menand *et al.*, 2007; Tam *et al.*, 2015). In Arabidopsis, *AtLRL1*, *AtLRL2*, and *AtLRL3* all promote root hair elongation and are functionally redundant (Breuninger *et al.*, 2016). In maize, *ZmLRL5* was highly expressed in elongating root hairs, where maize *zmlrl5* mutants exhibited shorter root hairs, indicating that *ZmLRL5* is critical for tip growth (Wang *et al.*, 2019). Interestingly, maize *zmlrl5* mutants had severely impaired ribosomal translation, and root hairs were highly sensitive to translation inhibition (Wang *et al.*, 2019). The ZmLRL5 protein regulates expression of ribosomal genes by binding to promoter regions, highlighting that continuous protein synthesis is critical for root hair growth (Wang *et al.*, 2019). Genes relating to ROS production were also found to be disrupted in the *zmlrl5* mutant, indicating that *ZmLRL5* probably acts to either directly or indirectly regulate ROS genes in addition to ribosomal genes (Wang *et al.*, 2019) (Fig. 4, pink).

### Maize summary

Root hair elongation in maize involves identical biological processes to those characterized in rice, including cell wall organization (*ZmRTH1*, *3*, and *6*), cell wall modification (*ZmRTH5*), calcium signalling (*ZmTIP1* and *ZmCPK9*), and nuclear regulation (*ZmLRL5*). Due to the relatively close evolutionary relationship between rice and maize, it is likely that while fewer root hair genes have been characterized in maize to date, further work will continue to identify additional maize orthologues of functionally characterized rice root hair genes.

## Wheat (*Triticum aestivum*)

The bread wheat genome is considerably larger [17 Gb, International Wheat Genome Sequencing Consortium (IWGSC), 2014] and more complex (hexaploid, consisting of A, B, and D subgenomes) than those of rice (450 Mb, diploid, Kurata *et al.*, 2002) and maize (2.4 Gb, diploid, Haberer *et al.*, 2005). Further, the presence of functional gene homoeologues in the A, B, and D subgenomes hinders mutant identification. Accordingly, molecular identification and characterization of root hair genes is more challenging in wheat than in species with diploid genomes. Accordingly, few studies have characterized root hair-related genes in wheat to date. Here, we summarize the genes currently known to be or implicated in the regulation of wheat root hair development, extending this to consider recent single cell expression datasets that allow identification of root hair-specific genes, many of which represent homologues of functionally validated Arabidopsis genes.

### Nuclear transcriptional regulation

Building on knowledge of the role of the Arabidopsis Class II bHLH transcription factor gene (*AtRSL4*) in root hair elongation, gene expression of a wheat homologue *TaRSL4* has been recently found to be positively correlated with wheat root hair length, with the A homoeologue showing increased expression compared with the B and D homoeologues across a panel of synthetic and natural wheat accessions (Han *et al.*, 2016). Transgenic overexpression of *TaRSL4-A* resulted in increased root hair length, supporting the role of *TaRSL4* in wheat root hair elongation. In a separate study, *TaRSL4* was found to be highly expressed in rainfed wheat cultivars under drought conditions, potentially indicating that *TaRSL4* is important for root hair elongation by acting as part of a drought tolerance mechanism under water stress (Maqbool *et al.*, 2022).

Similarly, gene expression of *TaRSL2*, a wheat homologue of the Arabidopsis Class I bHLH transcripton factor gene *AtRSL2*, is positively correlated with root hair length across a panel of synthetic and natural wheat accessions (Han *et al.*, 2017). Overexpression of the *TaRSL2*-*D* homoeologue in Arabidopsis increased root hair length, indicating that RSL2 function has been conserved across 200 million years (Wolfe *et al.*, 1989) since the divergence of monocot and dicot species (Han *et al.*, 2017). Interestingly, *TaRSL2-D* homoeologue overexpression in Arabidopsis also increased shoot biomass, with the authors proposing that this was a result of increased nutrient scavenging (Han *et al.*, 2017).

**Fig. 5.**
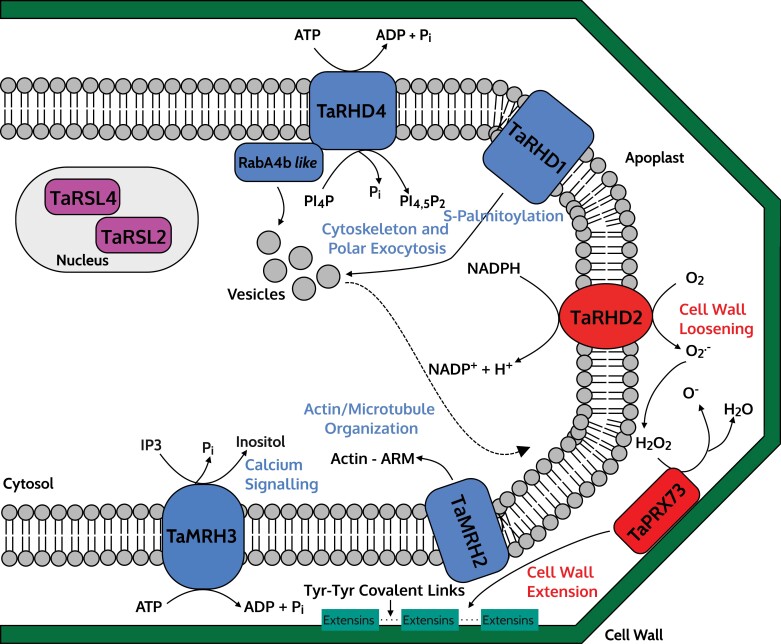
Molecular model for the control of root hair elongation in a wheat (*Triticum aestivum*) root hair cell (trichoblast). Solid lines indicate regulation, dashed lines indicate movement, and dotted lines indicate putative interactions. See main text and Table 5 for more details and references.

Expression biases between the A/D homoeologues was also detected in *TaRSL2*, with the B-homoeologue expressed at a lower level (Han *et al.*, 2017). In Arabidopsis, both *AtRSL4* and *AtRSL2* are critical regulators of root hair elongation. *AtRSL4* primarily regulates ROS production in developing trichoblasts (Mangano *et al.*, 2017; Marzol *et al.*, 2022), while *AtRSL2* primarily regulates the response to phosphate deficiency (Bhosale *et al.*, 2018). Despite strong evidence via conservation of gene sequence, gene expression, and interspecies transformation studies, the molecular functions of *TaRSL4* and *TaRSL2* have yet to be directly confirmed in wheat (Fig. 5, pink).

**Table 5.** Wheat (*Triticum aestivum* L.) genes likely to be involved in root hair elongation

Assigned gene name	Arabidopsis gene ID	Wheat homoeologues	Functional annotation	Reference
*TaRHD1* ^*a*^	AT5G20350	*TraesCS5A02G183100*	*S*-Acyltransferase	Hemsley *et al.* (2005)
		*TraesCS5B02G181300*		
		*TraesCS5D02G188200*		
*TaRHD2* ^*a*^	AT5G51060	*TraesCS3A02G354200*	RBOH	Kuběnová *et al.* (2022)
		*TraesCS3B02G386600*		
		*TraesCS3D02G347900*		
*TaRHD4* ^*a*^	AT3G51460	*TraesCS1A02G020700*	PI(4)P phosphatase	Thole *et al.* (2008)
		*TraesCS1B02G024700*		
		*TraesCS1D02G020200*		
*TaMRH2* ^*a*^	AT3G54870	*TraesCS4A02G036800*	Kinesin motor protein	Yang *et al.* (2007)
		*TraesCS4B02G269800*		
		*TraesCS7A02G076600*		
*TaMRH3* ^*a*^	AT5G65090	*TraesCS6A02G302300*	IP3-5 phosphatase-like	Jones *et al.* (2006)
		*TraesCS6B02G331500*		
		*TraesCS6D02G281800*		
*TaPRX73* ^*a*^	AT5G67400	*TraesCS7A02G262000*	Class III peroxidase	Marzol *et al.* (2022)
		*TraesCS7B02G159300*		
		*TraesCS7D02G262400*		
*TaRSL2*	AT4G33880	*TraesCS4A02G047700*	bHLH transcription factor	Han *et al.* (2017)
		*TraesCS4B02G257200*		
		*TraesCS4D02G257100*		
*TaRSL4*	AT1G27740	*TraesCS2A02G194200*	bHLH transcription factor	Han *et al.* (2016)
		*TraesCS2B02G212700*		
		*TraesCS2D02G193700*		

^*a*^ Identified in the scRNA dataset (Zhang *et al*., 2023). Wheat gene models: IWGSC RefSeqv1.1.

### Mining gene expression data to identify genes potentially controlling wheat root hair development

Analysis of gene expression profiles in different wheat root cell types has recently identified a network of genes expressed in a wheat trichoblast through the use of single-cell RNA-seq (scRNA-seq) technology (Table 5) (Zhang *et al.*, 2023). The majority of these genes were homologues of known Arabidopsis root hair genes. However, since the function of these newly discovered genes has yet to be confirmed *in planta*, we can only hypothesize about their function. We summarize here the possible functional roles of these recently identified wheat genes by drawing on data from Arabidopsis.

### *S*-Palmitoylation

The phenotype of the Arabidopsis *rhd1* mutant (short, branched root hairs) is regulated by a gene called *AtTIP1*. Within the scRNA-seq dataset, wheat homologues of *AtTIP1* (*TraesCS5A02G183100*, *TraesCS5B02G181300*, and *TraesCS5D02G188200*) were found to be highly expressed in trichoblasts (Zhang *et al.*, 2023). *AtTIP1* encodes an *S*-acyltransferase that regulates palmitoylation (Hemsley *et al.*, 2005). As with the maize homologue *ZmTIP1* discussed above, *S*-acylation is an important process that regulates protein–membrane association and vesicular trafficking (Hemsley *et al.*, 2005) (Fig. 5, blue). Inhibition of TIP1-mediated acylation in wild-type Arabidopsis produces the *tip1* phenotype with short, branched root hairs (Hemsley *et al.*, 2005).

### Cytoskeleton and polar exocytosis

The Arabidopsis mutant *rhd4* has short root hairs which form random bulges (Thole *et al.*, 2008). The *AtRHD4* gene encodes a Sac1p-like phosphoinositide (PI) phosphatase. PI phosphatases (and PI kinases) convert PIs (lipid molecules) between phosphorylation states for various cellular processes (Thole *et al.*, 2008; Hsu and Mao, 2015). The AtRHD4 protein localizes with its substrate RabA4b (a plant Rab GTPase) in the tips of root hairs (Fig. 5, blue). The latter regulates cargo membrane trafficking (and subsequent polar exocytosis) via recruiting cytosolic proteins (Thole *et al.*, 2008). Arabidopsis *rhd4* mutants have an altered actin cytoskeleton, with thinner and patchier filaments in epidermal cells compared with the wild type (Thole *et al.*, 2008). As a result, the spatial distribution of AtRabA4b is also disrupted in *rhd4* root hairs, where the fluctuation of localization of AtRabA4b in the root hair tip was correlated with elongation and bulge formation (Thole *et al.*, 2008). The wheat homologues of *AtRHD4* are *TraesCS1A02G020700*, *TraesCS1B02G024700*, and *TraesCS1D02G020200*.

The Arabidopsis *mrh2* mutant produces bulbous root hairs (Yang *et al.*, 2007). *AtMRH2* (MORPHOGENESIS OF ROOT HAIR 2) encodes a kinesin with a motor domain which is active throughout root hair elongation. Loss of function in *AtMRH2* results in randomly oriented microtubules within the root hair, leading to the formation of random bulges instead of wild-type root hair elongation (Yang *et al.*, 2007). AtMRH2 contains an Armadillo (ARM) domain that directly binds to polymerized actin within the cell, further implicating the role of *AtMRH2* in cytoskeleton organization and subsequent root hair elongation (Yang *et al.*, 2007) (Fig. 5, blue). Wheat homologues of *AtMRH2* are *TraesCS4A02G036800*, *TraesCS4B02G269800*, and *TraesCS7A02G076600*. Interestingly, elongating wheat root hairs have previously been shown to have increased amounts of G-actin (the subunit of F-actin; F-actin strands form microfilaments) at the cell apex, indicating that actin deposition is critical for root hair elongation. Furthermore, the chemical disruption of intracellular Ca^2+^ arrested the formation of the G-actin gradient in elongating wheat root hairs, highlighting the importance of Ca^2+^ signalling in regulating wheat cytoskeleton organization and root hair growth (He *et al.*, 2006).

### Calcium signalling

Arabidopsis *AtMRH3* function was identified by Jones *et al*. (2006) using a T-DNA insertion mutant of a candidate gene identified by Berdy *et al*. (2001). The *atmrh3* mutant produces straight root hairs with ballooned bases, indicating that *AtMRH3* is necessary for controlling root hair width. *AtMRH3* encodes an IP3 5-phosphatase (lipid raft protein), which regulates the IP3 signalling pathway by dephosphorylating the 5' phosphate group in IP3 to inositol, terminating the signalling pathway (Berdy *et al.*, 2001; Jones *et al.*, 2006). Termination of this pathway prevents IP3 (and other secondary messengers) from altering intracellular Ca^2+^ levels, affecting downstream developmental processes, such as cytoskeleton formation (Berdy *et al.*, 2001; Jones *et al.*, 2006) (Fig. 5, blue). Wheat homologues of *AtMRH3* are *TraesCS6A02G302300*, *TraesCS6B02G331500*, and *TraesCS6D02G281800*, whose products are all predicted to contain the same highly conserved endonuclease/exonuclease/phosphatase Pfam protein domain as found in AtMRH3. Recently, a wheat TILLING mutant (*stumpy*) producing long root hairs when grown in high Ca^2+^ soils has been identified (Zeng *et al.*, 2024). The *stumpy* candidate gene (*TraesCS7B03G0323100*) is predicted to encode an uncharacterized transmembrane protein, likely to be associated with Ca^2+^ signalling (Zeng *et al.*, 2024). The *stumpy* mutant (and other emerging root hair mutants) will probably provide entry points into future morphological studies of wheat root hair development.

### ROS production and cell wall modification

The role of ROS production from RBOHs in root hair elongation has been detailed earlier in this review. Mutation in an *RBOHC* gene underlies the *rhd2* phenotype in Arabidopsis, resulting in short root hairs that fail to elongate (Kuběnová *et al.*, 2022). Interestingly, Kuběnová *et al.* (2022) found that in Arabidopsis, endosomes (intracellular vesicles) carry AtRHD2 (ROOT HAIR DEFECTIVE 2) to the plasma membrane of the developing root hair apex; this process is negatively affected in *rhd2*. The wheat homologues of *AtRHD2* are *TraesCS3A02G354200*, *TraesCS3B02G386600*, and *TraesCS3D02G347900* (Fig. 5, red).

In Arabidopsis, *AtPRX73* encodes a Class III PER, responsible for ROS production and cell wall loosening/polymerization (Marzol *et al.*, 2022). The wheat homologues (*TraesCS7A02G262000*, *TraesCS7B02G159300*, and *TraesCS7D02G262400*) of Arabidopsis *AtPRX73* were found to be expressed in developing trichoblasts (Zhang *et al.*, 2023) (Fig. 5, red). Plant cell walls contain numerous cell wall modification enzymes, including PERs, expansins, and EXTs. EXTs are a part of the hydroxyproline-rich glycoprotein superfamily, which form critical networks for regulating cell wall development and polar expansion of root hairs and pollen tubes (Sede *et al.*, 2018; Marzol *et al.*, 2022). EXTs require post-translational modifications for proper function, including hydroxylation (addition of an -OH group via oxidation) and *O*-glycosylation [addition of a sugar (*O*-GlcNAc) to serine/threonine residues (Bektas and Rubenstein, 2011)], before being deposited in the cell wall and made insoluble (Marzol *et al.*, 2022). *AtPRX73*, along with *AtPRX01* and *AtPRX44*, are predicted to trigger cell wall extension via facilitating the formation of tyrosine–tyrosine covalent links between EXTs, promoting root hair growth (Marzol *et al.*, 2022) (Fig. 5, green). The Arabidopsis *atprx01 atprx44 atprx73* triple mutant displays a significant reduction in root hair ROS levels, as well as reduced cell wall thickness in root hair tips (Marzol *et al.*, 2022), indicating the role of PERs and EXTs in developing sufficiently thick root hairs.

## Conclusion

Changes in future agricultural environments, due to factors such as the effects of climate change and the drive for more environmentally sustainable food production practices, will result in new challenges for crop breeding. With renewed focus on below-ground crop phenotypes (Ober *et al.*, 2021), the informed modulation of root hair traits will probably play an increasing role in the breeding strategies to help optimize crop yield and resilience. Here, we provide the first review of the key genes controlling root hair development in the world’s most important crops: rice, maize, and wheat. Root hair development relies on many biological processes, including cell wall modification, ROS production, nuclear regulation, *S*-acylation, cytoskeleton organization, vesicular transport, and hormonal regulation. These processes are each regulated by complex gene networks, with significant crosstalk and interactions between other root hair developmental pathways. As evident from this review, the genes and gene functions regulating many of these processes are highly conserved across the monocot and dicot divide, controlling processes including nuclear regulation, cell wall modification, and cytoskeleton organization.

However, while many of the genes regulating root hair development in Arabidopsis root epidermal cell differentiation and root hair bulge initiation have been identified, very few genes controlling these processes have been identified in rice, maize, and wheat to date. The evidence presented here highlights good conservation of gene function between Arabidopsis and cereal crop species, which indicates that rapid progress in understanding the molecular genetic control of root hair development should be possible in these and other cereal crops. Ultimately, the availability of new genomic techniques such as transgene-free gene editing, combined with traditional plant breeding approaches, will allow the quantitative modulation of crop root hair morphology for best adaptation to future agricultural environments. To help achieve these goals, detailed investigation of the effects of the impact of different root hair architectures (e.g. variation in root hair length, density, and patterning) on nutrient scavenging, yield, and overall plant health will be required. Furthermore, there is significant scope to investigate the interplay between crop root hair morphology and other key root traits, such as root biomass and root branching. Since our understanding of how various root hair architecture traits influence crop performance in future agricultural conditions is sparse, we suggest exploring variation in root hair genes conserved across the monocot/dicot divide, such as *OsNOX3/ZmRTH5/TaRHD2*, which controls root hair initiation and elongation. Crop varieties exhibiting elevated expression of these genes and/or optimized protein function are likely to display longer, denser root hairs, which could be included in a package of root traits better adapted to current and future agricultural environments by increasing water uptake, nitrogen acquisition (Saengwilai *et al.*, 2021), phosphorus acquisition (Gahoonia and Nielsen, 1998; Ma *et al.*, 2001) and grain yield (Gahoonia and Nielsen, 2004). Given the underexplored nature of crop root hair traits (Table 1), future crop breeding efforts should integrate the optimization of root hair architectures as a key trait for crop improvement.
